# Supplementary material for: Overexpression of NOX2 Exacerbates AngII-Mediated Cardiac Dysfunction and Metabolic Remodelling
Source: Antioxidants (Basel). 2022 Jan 10;11(1):143. doi: 10.3390/antiox11010143 (PMC8772838; doi:10.3390/antiox11010143)
Supplement: Supplementary file 1 [file antioxidants-11-00143-s001.zip › antioxidants-1514988-supplementary.pdf]

antioxidants

MDPI

Supplemented data

Preparation of homogenate: Frozen heart tissue was toughed in ice-cold A1 buffer (250 mM Sucrose, 0.5 mM Na_2EDTA, 10 mM Tris, pH 7.4). The following steps were performed on ice. Approximately 20 mg tissue in 0.5 mL A1 buffer was cut into 1x1 mm pieces. After 10 min incubation at 4°C with 0.5 mg/mL trypsin (Sigma Aldrich), the buffer was removed, and the sample was washed twice with 2 mL A1. Thereafter, the tissue was transferred to a shredder tube (Oroboros Instrument, Innsbruck, Austria) containing 0.5 mL Mir05 (0.5 mM EGTA, 3 mM $MgCl_2x6H_2O$, 20 mM Taurine, 10 mM KH_2PO_4, 20 mM HEPES, 110 mM Sucrose, 50 mM K-lactobionate, 1 g/L BSA, pH 7.1). Tissue homogenate was made by placing the shredder tube in a shredder holder (Oroboros Instrument) and homogenize for 12 sec. at position 1 by using the PBI-Shredder SG3 (Oroboros Instrument). The homogenate was collected and centrifuged (Heraeus Biofuge, Thermo Scientific) at appr. 60 x g in 1 min. Finally, the supernatant was transferred to an Eppendorf tube and stored on ice until respiration analysis.

Table S1. mRNA expression of genes in cardiac tissue from C57BL/6J mice treated with 50ng/kg/min AngII ($AngII_{50}$) or saline (sham) for two weeks.

	**Sham**	**$AngII_{50}$**
n	***7***	***10***
iNOS	1 ± 0.15	0,7 ± 0.08
ucp3	1 ± 0.11	1.1 ± 0.17
nppb	1.0 ± 0.1	1.2 ± 0.2
nppa	1.0 ± 0.1	1.3 ± 0.2

Data are means ± SEM. *nppb*, natriuretic peptide b; *nppa*, natriuretic peptide A; iNOS, inducible nitric oxide synthase; *ucp3*, uncoupling protein 3. mRNA expression of the genes of interest was adjusted to the housekeeping gene hypoxanthine guanine phosphoribosyl transferase *hprt.*

Table S2. mRNA expression of genes in cardiac tissue from C57BL/6J mice treated with 400 ng/kg/min AngII ($AngII_{400}$) or saline (sham) for two weeks.

	**Sham**	**$AngII_{400}$**
n	***9-10***	***9-10***
pparα	1.00 ± 0.12	0.97 ± 0.05
cd36	1.00 ± 0.06	0.96 ± 0.03
pdk4	1.00 ± 0.13	0.73 ± 0.08
ldh	1.00 ± 0.04	0.87 ± 0.03#
hk	1.00 ± 0.03	0.96 ± 0.05
nox2	1.00 ± 0.09	1.10 ± 0.11

Data are means ± SEM. *pparα*, peroxisome proliferator-activated receptor *α*; *cd36*, cluster of differentiation 36; *pdk4*, pyruvate dehydrogenase kinase 4; *ldh*, lactate dehydrogenase; *hk*, hexokinase; *nox2*, nicotinamide-adenine dinucleotide phosphate oxidase 2. # $p < 0.05$ compared to sham. mRNA expression of the genes of interest was adjusted to the housekeeping gene hypoxanthine guanine phosphoribosyl transferase *hprt*

Table S3. mRNA expression of genes in cardiac tissue from male (WT) and cs NOX2 transgenic (TG) mice treated with 400 ng/kg/min AngII ($AngII_{400}$) for two weeks.

	**WT $AngII_{400}$**	**TG $AngII_{400}$**
n	***4-6***	***5-6***
nppb	1.00 ± 0.11	0.86 ± 0.10
nppa	1.00 ± 0.20	0.72 ± 0.22
pparα	1.00 ± 0.05	0.97 ± 0.03
cd36	1.00 ± 0.04	0.98 ± 0.02
pdk4	1.00 ± 0.07	1.57 ± 0.13[#]
ldh	1.00 ± 0.05	0.93 ± 0.04
hk	1.00 ± 0.02	1.00 ± 0.02
nox2	1.00 ± 0.06	33.51 ± 1.08 [#]

Data are means ± SEM. *nppb*, natriuretic peptide b; *nppa*, natriuretic peptide A; *pparα*, peroxisome proliferator-activated receptor *α*; *cd36*, cluster of differentiation 36; *pdk4*, pyruvate dehydrogenase kinase 4; *ldh*, lactate dehydrogenase; *hk*, hexokinase; *nox2*, nicotinamide-adenine dinucleotide phosphate oxidase 2. mRNA expression of the genes of interest was adjusted to the housekeeping gene hypoxanthine guanine phosphoribosyl transferase *hprt*. # $p < 0.05$ vs WT.

Table S4.
mRNA expression of the genes of interest was adjusted to the housekeeping gene hypoxanthine guanine phosphoribosyl transferase *hprt*.

	**Gene Forward**	**Gene Accession Number**	**Reverse**
iNOS	CAG-CTG-GGC-TGT-ACA	NM_010927.4	CAT-TGG-AAG-TGA-AGC-GTT
ucp3	TAC-CCA-ACC-TTG-GCT-AGA	NM_009464.3	GCC-TGG-CAA-TCT-TTT-GCT-T
nppb	CCA GTC TCC AGA GCA ATT CAA	NM_008726.5	GCC ATT TCC TCC GAC TTT T
nppa	CAC AGA TCT GAT GGA TTT CAA GA	NM_008725.3	CCT CAT CTT CTA CCG GCA TC
pparα	ACG ATG CTG TCC TCC TTG ATG	NM_0111446	GTG TGA TAA AGC CAT TGC CGT
cd36	TTG TAC CTA TAC TGT GGC TAA ATG AGA	NM_001159558.1	CTT GTG TTT TGA ACA TTT CTG CTT
pdk4	TTC ACA CCT TCA CCA CAT GC	NM_013743.2	AAA GGG CGG TTT TCT TGA TG
ldh	CAT TGT CAA GTA CAG TCC ACA CT	NM_010699.2	TTC CAA TTA CTC GGT TTT TGG GA
hk	GAA GGG GCT AGG AGC TAC CA	NM_013820.3	CTC GGA GCA CAC GGA AGT T
nox2	TGAATGCCAGAGTCGGGATTT	NM_007807.5	CCCCCTTCAGGGTTCTTGATTT
hprt	TCC TCC TCA GAC CGC TTT T	NM_013556.2	CCT GGT TCA TCA TCG CTA ATC

Inducible nitric oxide synthase (*iNOS*); uncoupling protein 3 (*ucp3*); natriuretic peptide B(*nppb*); natriuretic peptide A (*nppa*); peroxisome proliferator-activated receptor *α* (*pparα*); cluster of differentiation 36 (*cd36*); pyruvate dehydrogenase kinase 4 (*pdk4*); lactate dehydrogenase (*ldh*); hexokinase (*hk*); nicotinamide-adenine dinucleotide phosphate oxidase 2 (*nox2*); hypoxanthine guanine phosphoribosyl transferase *hprt*.

Table S5. *Ex vivo* steady-state measurements of cardiac function from C57Bl/6J, WT and csNOX2 transgenic (TG) mice treated for two weeks with micro-osmotic pumps containing either Saline (sham), 50 or 400ng/kg/min Angiotensin II ($AngII_{50}$ and $AngII_{400}$). The data are presented as mean ± SEM.

	Sham *n* = 9	$AngII_{50}$ *n* = 11	Sham *n* = 6	$AngII_{400}$ *n* = 9	WT $AngII_{400}$ *n* = 5	TG $AngII_{400}$ *n* = 6
**BPM**	**389 ± 12**	**384 ± 10**	**349 ± 17**	**361 ± 13**	**361 ± 17**	**324 ± 16**
**CO (mL/min)**	**13.5 ± 0.7**	**12.2 ± 0.6**	**13 ± 1**	**13 ± 1**	**13.0 ± 0.9**	**13.4 ± 0.6**
**CF (mL/min)**	**2.8 ± 0.2**	**3.5 ± 0.2**	**3.4 ± 0.3**	**3.2 ± 0.2**	**4.0 ± 0.5**	**3.8 ± 0.3**
**DEVP (mmHg)**	**37 ± 2**	**37 ± 3**	**42 ± 3**	**41 ± 3**	**45 ± 3**	**52 ± 3**
**dp/dt_{max} (mmHg/s)**	**1641± 85**	**1615 ± 106**	**1611± 110**	**1663 ± 84**	**1940 ± 48**	**1888 ± 62**
**dp/dt_{min} (mmHg/s)**	**-844 ± 93**	**-950 ± 150**	**-821 ± 114**	**-718 ± 146**	**-1024 ± 96**	**-1008 ± 86**

BPM beats per minute; CO, Cardiac Output; CF, Coronary Flow; DEVP, Aortic developed pressure; dp/dt_{max} and dp/dt_{min}, maximal and minimal first derivative of pressure over time, respectively.
